# Code-Free AutoML for Binary Classification of Fractured and Non-fractured Bone Radiographs From a Heterogeneous Public Dataset Using Google Cloud Vertex AI: A Proof-of-Concept Study

**DOI:** 10.7759/cureus.115047

**Published:** 2026-08-23

**Authors:** Mark A Bachir, Neel Nawathey, Alexander Bachir, Akshay J Reddy, Jasmeen K Cheema, Rakesh Patel

**Affiliations:** 1 Internal Medicine, California Northstate University College of Medicine, Elk Grove, USA; 2 Osteopathic Medicine, Touro University California, Vallejo, USA; 3 Medicine, California Health Sciences University, Clovis, USA; 4 Medicine, California University of Science and Medicine, Colton, USA; 5 Medicine, University of California, Davis, Davis, USA; 6 Internal Medicine, East Tennessee State University Quillen College of Medicine, Johnson City, USA

**Keywords:** artificial intelligence, automl, bone fracture, computer vision, deep learning, fracture detection, image classification, musculoskeletal radiology, radiography, vertex ai

## Abstract

Fracture detection on radiographs can be challenging, particularly for subtle or nondisplaced injuries, while development of artificial intelligence (AI) models often requires programming expertise and specialized computational resources. This proof-of-concept study evaluated whether a commercially available code-free automated machine-learning platform could distinguish fractured from non-fractured bone radiographs.

A publicly available heterogeneous dataset containing 420 radiographic images, including 130 labeled as fractured and 290 labeled as non-fractured, was imported into Google Cloud Vertex AI AutoML Image Classification (Google, Inc., Mountain View, CA, USA) and divided into 336 training, 42 validation, and 42 test images. The dataset included radiographs from multiple anatomical regions; however, the publicly available metadata did not provide a sufficiently detailed anatomical distribution to reliably quantify upper-extremity, lower-extremity, spinal, or other fracture categories. Importantly, these counts represent radiographic images rather than confirmed independent patients, and patient-level distribution and independence across the training, validation, and test subsets could not be verified. No additional preprocessing, augmentation, or relabeling was performed by the investigators. Model performance was assessed using average precision, platform-reported aggregate precision and recall, confidence-threshold analysis, and a row-normalized confusion matrix.

The trained classifier achieved a micro-averaged area under the precision-recall curve (AuPRC), reported by Vertex AI as average precision, of 0.987. At a confidence threshold of 0.50, model-level precision and recall were each 90.5%. Because these values summarize performance across both classes, they should not be interpreted as fracture-specific sensitivity. Class-specific average precision was 0.994 for non-fractured images and 0.972 for fractured images, while fracture-class recall was 77%. The row-normalized confusion matrix demonstrated correct classification of 77% of fractured radiographs and 97% of non-fractured radiographs. However, the retained Vertex AI output provided normalized percentages rather than raw class counts; therefore, the absolute numbers of correctly and incorrectly classified fractured and non-fractured images could not be verified. Given the small 42-image test set, the absence of raw counts limits the precision, interpretability, and reproducibility of these class-specific performance estimates and should be considered an important methodological limitation. The model was also successfully deployed to an online prediction endpoint and generated high-confidence classifications for representative images.

These findings demonstrate that code-free AutoML can support rapid development and deployment of a functional, internally evaluated fracture-classification model without custom programming. However, the 77% fracture-class recall and small 42-image test set indicate that the model’s performance should be interpreted cautiously and not as evidence of clinical diagnostic effectiveness. The small test set, class imbalance, limited clinical metadata, uncertain patient-level independence, and absence of external validation limit generalizability. Code-free AutoML may therefore be useful for proof-of-concept medical imaging research and education, but external validation is required before clinical implementation.

## Introduction

Fractures are among the most common traumatic musculoskeletal injuries and account for a substantial proportion of emergency department visits, orthopedic consultations, and trauma-related hospital admissions. Prompt and accurate diagnosis is important because missed or delayed fractures may result in prolonged pain, impaired mobility, delayed union, nonunion, unnecessary disability, and avoidable intervention. Conventional radiography remains the first-line imaging modality for suspected fractures because it is widely available, rapidly acquired, relatively inexpensive, and generally associated with lower radiation exposure than computed tomography [1]. Nevertheless, radiographic interpretation can be challenging in cases involving subtle, nondisplaced, stress, or occult fractures and in anatomically complex regions where overlapping structures may obscure fracture lines. These diagnostic challenges are compounded by variability in reader experience, workload, fatigue, image quality, and clinical context, all of which can affect the consistency of radiographic interpretation [2]. These limitations have contributed to growing interest in artificial intelligence (AI) as a potential adjunct for fracture detection and radiographic decision support.

AI has emerged as a potential adjunct to medical image interpretation, with deep-learning systems demonstrating promising performance across numerous radiologic applications, including musculoskeletal imaging [3-10]. Prior studies suggest that AI-assisted fracture detection can support clinician interpretation and, in controlled settings, may improve fracture-detection performance; however, evidence regarding effects on workflow efficiency, interpretation time, and patient outcomes remains limited [3-5]. Systematic reviews and meta-analyses have reported strong diagnostic performance for AI models across several anatomical regions while emphasizing that favorable internal validation results do not necessarily translate to external clinical environments [3,4]. Accordingly, these systems are best considered potential decision-support tools rather than replacements for clinical judgment.

Traditional deep-learning development commonly requires programming expertise, familiarity with machine-learning frameworks, hyperparameter optimization, specialized computational hardware, and software-engineering experience. These requirements may limit participation by clinicians, medical students, and biomedical researchers interested in medical AI. Automated machine-learning platforms can reduce technical barriers associated with model development by automating tasks such as image preprocessing, architecture selection, hyperparameter optimization, training, evaluation, and deployment through graphical interfaces requiring little or no custom programming [11-16]. However, code-free implementation does not eliminate methodological complexity; rigorous dataset curation, prevention of data leakage, appropriate class definition, validation, calibration, and clinical evaluation remain essential components of reliable medical AI research. Previous studies have shown that code-free AutoML platforms can achieve strong classification performance in selected medical imaging tasks, including ophthalmic and multimodality image classification, when evaluated on task-specific datasets and metrics [13-16]. However, performance varies substantially according to dataset characteristics, class definition, validation strategy, and evaluation methodology, limiting direct comparison across studies.

Medical AI models may demonstrate favorable performance during internal evaluation yet fail to generalize when applied to images from different institutions, imaging systems, or patient populations [17]. This concern is particularly important for studies using heterogeneous public datasets, in which incomplete information regarding image provenance, anatomical distribution, patient-level independence, labeling procedures, and data partitioning may limit interpretation of model performance. Reporting frameworks such as the Checklist for Artificial Intelligence in Medical Imaging and TRIPOD+AI emphasize transparent documentation of dataset provenance, outcome definition, model development, validation procedures, evaluation metrics, and uncertainty [17,18]. In the present study, several of these elements could not be fully characterized because of limitations in the publicly available dataset and the proprietary AutoML platform, including patient-level independence, detailed anatomical distribution, some training parameters, raw class-specific prediction counts, and external validation. These limitations are therefore considered important constraints on reproducibility and interpretation rather than being resolved by adherence to reporting guidelines alone.

Against this background, the objective of this study was to assess the technical feasibility and internal classification performance of a code-free binary image-classification workflow for distinguishing radiographs labeled as fractured from those labeled as non-fractured using Google Cloud Vertex AI AutoML Image Classification. The study was designed as a proof-of-concept evaluation of whether such a model could be developed and deployed without investigator-written code or manual neural-network design, rather than as an assessment of clinical diagnostic effectiveness.

## Materials and methods

Study design

This proof-of-concept study evaluated the feasibility of developing a binary image-classification model capable of distinguishing fractured from non-fractured bone radiographs using a commercially available code-free automated machine-learning platform. Model development was performed using Google Cloud Vertex AI AutoML Image Classification, which automates image preprocessing, neural-network architecture selection, hyperparameter optimization, training, evaluation, and deployment through a graphical interface requiring no custom programming. The study was designed to assess technical feasibility and internal model performance rather than clinical effectiveness [13-16].

Dataset

Bone radiographs were derived from the publicly available X-Ray Imaging Dataset for Detecting Fractured vs. Non-Fractured Bones, Version 3, hosted on Mendeley Data [17-21]. According to the source dataset documentation, the original collection consists of 420 human bone radiographic images obtained from two hospital laboratories, including 130 images labeled as fractured and 290 labeled as non-fractured. The fracture-status labels were clinically verified by a consultant surgeon identified by the dataset authors [21]. The public documentation does not provide the number of unique patients, number of radiographs per patient, detailed anatomical distribution, fracture subtype, imaging projection, patient age distribution, or criteria regarding inclusion of pathological fractures or postoperative images. Similarly, although images are categorized as fractured or non-fractured, more detailed diagnostic criteria defining the non-fractured class are not reported. Therefore, the 420 observations in this study should be interpreted as radiographic images rather than 420 independent patients. Patient-level duplication and independence across the training, validation, and test partitions could not be established from the available metadata, and detailed upper-extremity, lower-extremity, spinal, or other anatomical subgroup analyses were not possible. The Kaggle derivative was selected because it provided a machine-learning-ready version of the publicly available source dataset that could be directly imported into the AutoML workflow [19-22]. According to the derivative dataset documentation, image-level curation included removal of corrupted and duplicate files, conversion to JPEG format, resizing to 224 × 224 pixels, organization into fractured and non-fractured classes, and provision of predefined training, validation, and test subsets. No additional preprocessing, augmentation, relabeling, or image curation was performed by the investigators after downloading the derivative dataset. The original Mendeley Version 3 dataset contains 420 original radiographs, comprising 130 fractured and 290 non-fractured images, in addition to separately generated augmented images. However, we could not independently verify image-for-image identity between the Kaggle derivative and the original Mendeley files, nor could we determine from the available documentation whether the training, validation, and test partition was inherited directly from the original dataset or created by the Kaggle curator. These uncertainties are acknowledged as limitations to complete reproducibility. According to the derivative dataset documentation, corrupted files and image-level duplicates were removed, the radiographs were converted to JPEG format and resized to 224 × 224 pixels, and the images were organized into fractured and non-fractured categories with predefined training, validation, and test subsets. Importantly, removal of duplicate image files does not establish patient-level deduplication. Because patient identifiers and the number of radiographs per patient were not available, the investigators could not determine whether multiple radiographs from the same patient were present or whether patient-level independence was maintained across the training, validation, and test subsets.

The dataset contained 420 radiographic images, including 130 fractured images (31.0%) and 290 non-fractured images (69.0%), resulting in a substantial class imbalance. No investigator-directed class weighting, oversampling, undersampling, or other class-balancing procedure was performed. The documented Vertex AI AutoML image-classification configuration exposes settings such as data partitioning, training budget, single- versus multi-label classification, and early stopping, but does not provide user-controlled class-weighting or sampling parameters; therefore, whether the proprietary AutoML training process internally adjusted for class imbalance could not be determined from the available documentation. Because the class distribution may influence reported classification metrics, particularly those dependent on the relative frequency of positive and negative predictions, performance was interpreted alongside class-specific results rather than aggregate metrics alone. All denominators in this study refer to radiographic images, not confirmed independent patients. The predefined dataset split consisted of 336 training images, 42 validation images, and 42 test images. The source dataset comprised radiographic images collected from two hospital laboratories and categorized as fractured or non-fractured, with fracture-status labels clinically verified by a consultant surgeon. The publicly available metadata did not provide sufficient detail to determine the specific fracture types, anatomical regions, imaging projections, or patient-level characteristics represented; therefore, no particular fracture type or anatomical area was selectively chosen by the investigators, and subgroup analyses by upper extremity, lower extremity, spine, or other regions could not be performed. Images were imported into Google Cloud Vertex AI in their publicly distributed form, and no additional augmentation, relabeling, manual annotation, or image modification was performed by the investigators. Data analysis was conducted as a binary single-label classification task, with images categorized as fractured or non-fractured and model performance evaluated on the held-out 42-image test set using average precision, precision, recall, confidence-threshold analysis, and a row-normalized confusion matrix.

According to the original dataset documentation, fracture-status labels were clinically verified by a consultant surgeon; however, the documentation does not describe the adjudication protocol in sufficient detail to determine whether labels underwent independent radiologist review, multiple-reader consensus, or formal orthopedic adjudication. The investigators therefore accepted the publicly provided fracture and non-fracture labels without additional independent review. This limited information regarding the reference standard represents a potential source of label uncertainty.

Patient identifiers and the number of radiographs obtained per patient were not available in the public dataset metadata. Consequently, patient-level independence across the training, validation, and held-out test subsets could not be verified. Removal of duplicate image files does not establish patient-level deduplication, and multiple radiographs from the same patient could potentially have been distributed across different subsets. Such overlap could introduce data leakage by allowing the model to encounter patient-specific or examination-specific characteristics during both training and testing, potentially inflating the reported internal test performance. Accordingly, the inability to confirm patient-level separation represents a fundamental limitation of the present study and warrants cautious interpretation of all reported performance estimates.

Model development

Model development was performed using Google Cloud Vertex AI AutoML Image Classification (Google, Inc., Mountain View, CA, USA) [19-23]. Images were imported into a single-label image-classification dataset configured for binary classification because the source dataset provided only two image-level labels: fractured and non-fractured. This configuration was therefore consistent with the available reference labels but represents a simplified formulation of the clinical problem. The publicly available metadata did not provide sufficiently detailed information regarding anatomical region, number of fractures per image, or additional abnormalities; consequently, the model was not designed to distinguish among fracture types, anatomical locations, or coexisting findings. Its output should therefore be interpreted only as binary image-level classification rather than comprehensive fracture detection or characterization. The AutoML platform automatically performed image preprocessing, feature extraction, neural-network architecture selection, hyperparameter optimization, and model training using managed cloud infrastructure.

Training was performed using Google Cloud Vertex AI AutoML Image Classification through the Google Cloud console in August 2026 [23]. The model was configured for cloud-hosted, single-label image classification with a maximum investigator-specified training budget of eight node-hours. The predefined dataset partition consisted of 336 training images (80%), 42 validation images (10%), and 42 held-out test images (10%). Vertex AI AutoML supports early stopping by default unless explicitly disabled; early stopping was not disabled in this study. No investigator-directed data augmentation, class weighting, resampling, optimizer selection, learning-rate scheduling, batch-size selection, or loss-function modification was performed. The proprietary platform did not expose the final neural-network architecture, internal optimization procedure, whether additional platform-level augmentation or class-imbalance handling was applied, or a user-visible model-version identifier beyond the AutoML image-classification service available at the time of training. These unavailable implementation details limit exact computational reproducibility and are acknowledged as a methodological limitation. Google Cloud documentation identifies an 80%/10%/10% training-validation-test allocation as the standard AutoML image-data split when automatic splitting is used, although the present study used the dataset’s predefined 336/42/42 partition. No investigator-directed modifications were made to the model architecture, optimizer, learning-rate schedule, batch size, transfer-learning parameters, or loss function. Because Vertex AI AutoML is a proprietary platform, the final neural-network architecture and several underlying training parameters were not disclosed and could not be reported. No custom programming was used during model development.

Model evaluation

Following training, model performance was evaluated on the platform-designated test set using metrics generated by Google Cloud Vertex AI. Average precision was reported because it was the principal threshold-independent discrimination metric generated by Google Cloud Vertex AI for the trained classifier, rather than because it was considered a superior clinical diagnostic metric. Average precision summarizes the precision-recall relationship across classification thresholds and is particularly informative in the presence of class imbalance; however, it is not equivalent to accuracy, sensitivity, specificity, or area under the receiver operating characteristic curve (AUROC). Accordingly, model performance was also interpreted using class-specific recall and the row-normalized confusion matrix at the 0.50 confidence threshold. Fracture-class recall was 77%, whereas non-fracture-class recall was 97%. Raw class-specific prediction counts were not available in the retained platform output; therefore, positive predictive value (PPV), negative predictive value (NPV), exact sensitivity and specificity confidence intervals, and other count-dependent diagnostic measures could not be reliably calculated. Given the small 42-image held-out test set, the absence of raw counts and 95% confidence intervals substantially limits the precision and clinical interpretability of the reported performance estimates. Additional evaluation metrics included model-level precision and recall at a confidence threshold of 0.50, class-specific average precision, precision-recall analysis, confidence-threshold analysis, and a row-normalized confusion matrix. The term “aggregate” was removed because the Vertex AI documentation does not explicitly characterize the threshold-specific precision and recall fields as macro-averaged or weighted averages. In contrast, the overall area under the precision-recall curve (AuPRC), reported as average precision, is explicitly defined by Google as micro-averaged for the overall evaluation. Class-specific results were therefore reported separately to avoid obscuring performance in the fracture class.

For the fracture class, recall represented the proportion of true fractured radiographs correctly classified by the model. The corresponding non-fracture row represented the proportion of true non-fractured radiographs correctly classified. The precision-recall and confidence-threshold curves were examined to characterize the tradeoff between false-positive and false-negative predictions. The row-normalized confusion matrix was reported using the percentages displayed by the AutoML platform. Raw per-class prediction counts and confidence intervals were not available in the retained evaluation output and were not reconstructed from rounded percentages.

Evaluation was limited to internal validation generated by the AutoML platform. No external institutional dataset, prospective clinical cohort, or clinician comparator was used. External validation using independent radiographic datasets would be required before consideration of clinical implementation [3,4,19,20].

Endpoint deployment

After model training, the selected classifier was temporarily deployed to a managed online prediction endpoint within Google Cloud Vertex AI. Four representative radiographs were submitted through the graphical interface to confirm that the deployed model could generate classification probabilities. These examples were used solely to demonstrate endpoint functionality and were not treated as an independent validation set or included in the reported performance estimates. The endpoint was removed after completion of the evaluation to minimize unnecessary cloud-computing costs.

Ethical considerations

This study used a publicly available anonymized dataset distributed for research and educational purposes under a Creative Commons Attribution 4.0 license [21]. The investigators had no access to identifiable patient information and did not interact with human participants. Because the study exclusively analyzed publicly available de-identified images, institutional review board approval and informed consent were not required.

Reporting standards

The manuscript was prepared with consideration of the Checklist for Artificial Intelligence in Medical Imaging and the TRIPOD+AI reporting guidelines to promote transparent documentation of the dataset, model-development process, validation methods, and performance measures [17,18].

Data availability

The original radiographic dataset is publicly available through Mendeley Data [21], and the derivative dataset used for model development is available through Kaggle [22]. No custom source code was generated because all model-development procedures were completed through the Google Cloud Vertex AI graphical interface.

## Results

Dataset characteristics

A total of 420 radiographic images were successfully imported into Google Cloud Vertex AI AutoML without import failures or image-format incompatibilities. The dataset included 130 fractured and 290 non-fractured radiographs and was divided into 336 training images, 42 validation images, and 42 test images.

Model training

Model training was completed using Google Cloud Vertex AI AutoML Image Classification with a maximum training budget of eight node-hours. The platform automatically performed image preprocessing, feature extraction, neural-network architecture selection, hyperparameter optimization, and model training without investigator modification of the model architecture or training parameters. Training was completed in approximately 1 hour and 48 minutes under the cloud infrastructure available during the study period. This duration is reported only as an operational observation and should not be interpreted as a reproducible model-performance outcome, because training time may vary with cloud resources, platform implementation, system load, and future software updates. No manual interruption or retraining was required.

Classification performance

**Figure 1:**
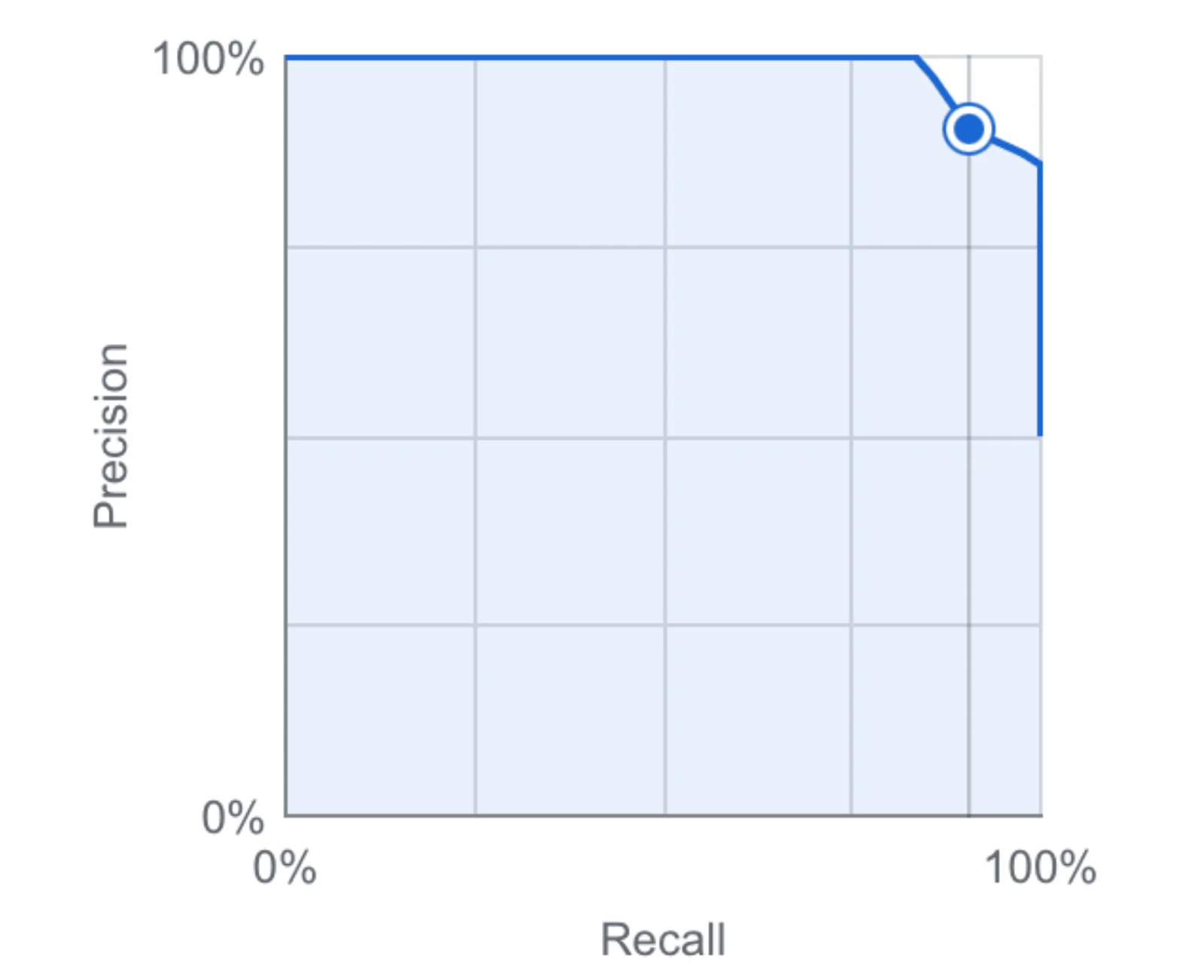
Precision-Recall Curve Generated by Google Cloud Vertex AI AutoML The precision-recall curve demonstrates classifier performance across multiple decision thresholds on the platform-designated test set. The model achieved an overall average precision of 0.987. Class-specific average precision was 0.994 for non-fractured radiographs and 0.972 for fractured radiographs, indicating strong internal discrimination between the two image classes.

At the 0.50 confidence threshold, the model’s performance was characterized using multiple complementary metrics rather than average precision alone. The overall average precision was 0.987; however, this threshold-independent metric should be interpreted cautiously given the small 42-image held-out test set, class imbalance, and uncertainty regarding patient-level independence. More clinically informative class-specific results showed 77% recall for fractured radiographs and 97% recall for non-fractured radiographs. Accordingly, the average precision value should not be interpreted as a general measure of clinical diagnostic performance. The platform-reported aggregate precision and recall were both 90.5%. Class-specific average precision was 0.994 for non-fractured radiographs and 0.972 for fractured radiographs. As shown in Figure 1, the precision-recall curve demonstrated strong overall discrimination between fractured and non-fractured images across a range of recall values.

Confidence-threshold analysis

**Figure 2:**
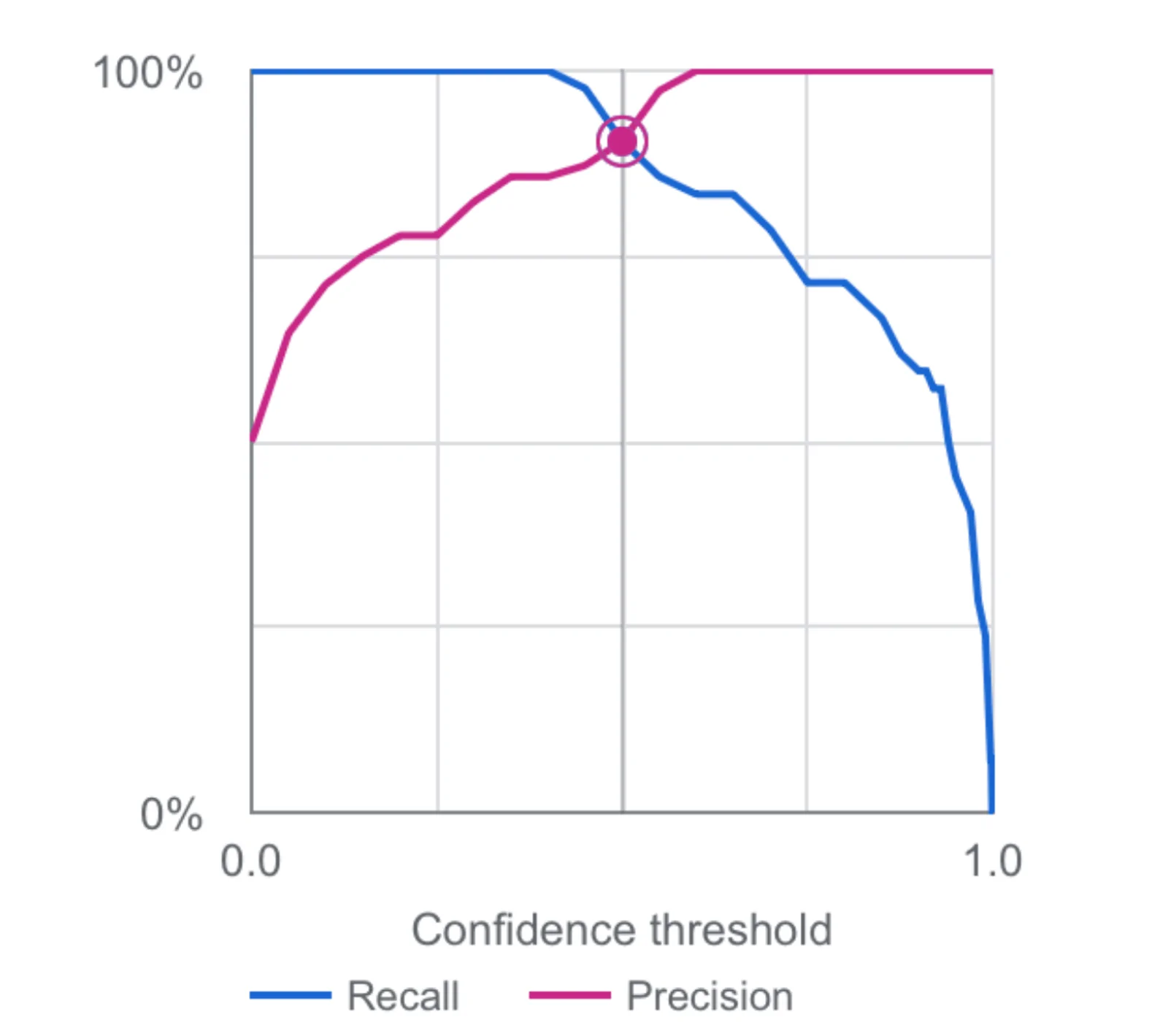
Precision and Recall Across Classification Confidence Thresholds Precision and recall are displayed across varying classification confidence thresholds. Increasing the threshold improved precision while reducing recall, demonstrating the expected trade-off between minimizing false-positive classifications and retaining fracture-detection sensitivity. The highlighted operating point represents the platform-selected threshold used during model evaluation.

Increasing the classification confidence threshold produced the expected tradeoff between precision and recall. As shown in Figure 2, precision progressively increased as the threshold increased, whereas recall declined. This relationship demonstrates that selecting a higher threshold may reduce false-positive classifications but may also increase the number of fractures incorrectly classified as non-fractured. The threshold was not optimized for clinical use.

Confusion-matrix analysis

**Figure 3:**
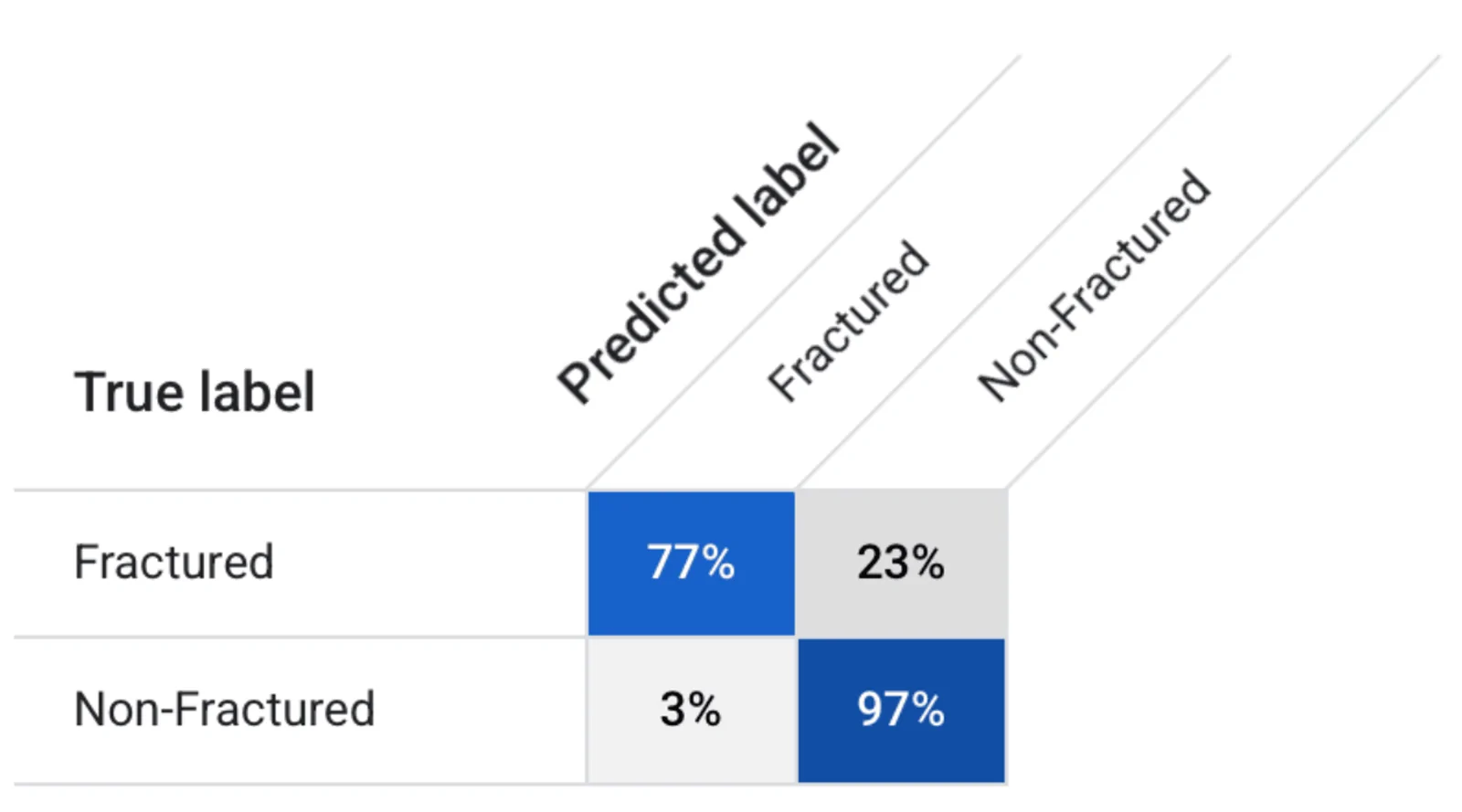
Row-Normalized Confusion Matrix for Fractured and Non-fractured Radiographs The row-normalized confusion matrix displays class-specific model performance at the default confidence threshold of 0.50. The model correctly classified 77% of fractured radiographs and 97% of non-fractured radiographs. Approximately 23% of fractured images were classified as non-fractured, whereas 3% of non-fractured images were classified as fractured. Values are presented as percentages rather than raw image counts.

Class-specific evaluation revealed asymmetric classification performance. As shown in Figure 3, the model correctly classified 77% of fractured radiographs and 97% of non-fractured radiographs. The retained Vertex AI output provided normalized percentages but not the corresponding raw class counts; therefore, the absolute numbers of correctly and incorrectly classified images could not be verified and were not reconstructed from rounded percentages. Approximately 23% of fractured radiographs were classified as non-fractured, compared with 3% of non-fractured radiographs classified as fractured. Given the small 42-image held-out test set, these percentages should be interpreted cautiously. Most importantly, the model failed to identify approximately one in four fractured images, indicating that its fracture-class performance is insufficient to support claims of clinical diagnostic usefulness.

Representative endpoint predictions

**Figure 4:**
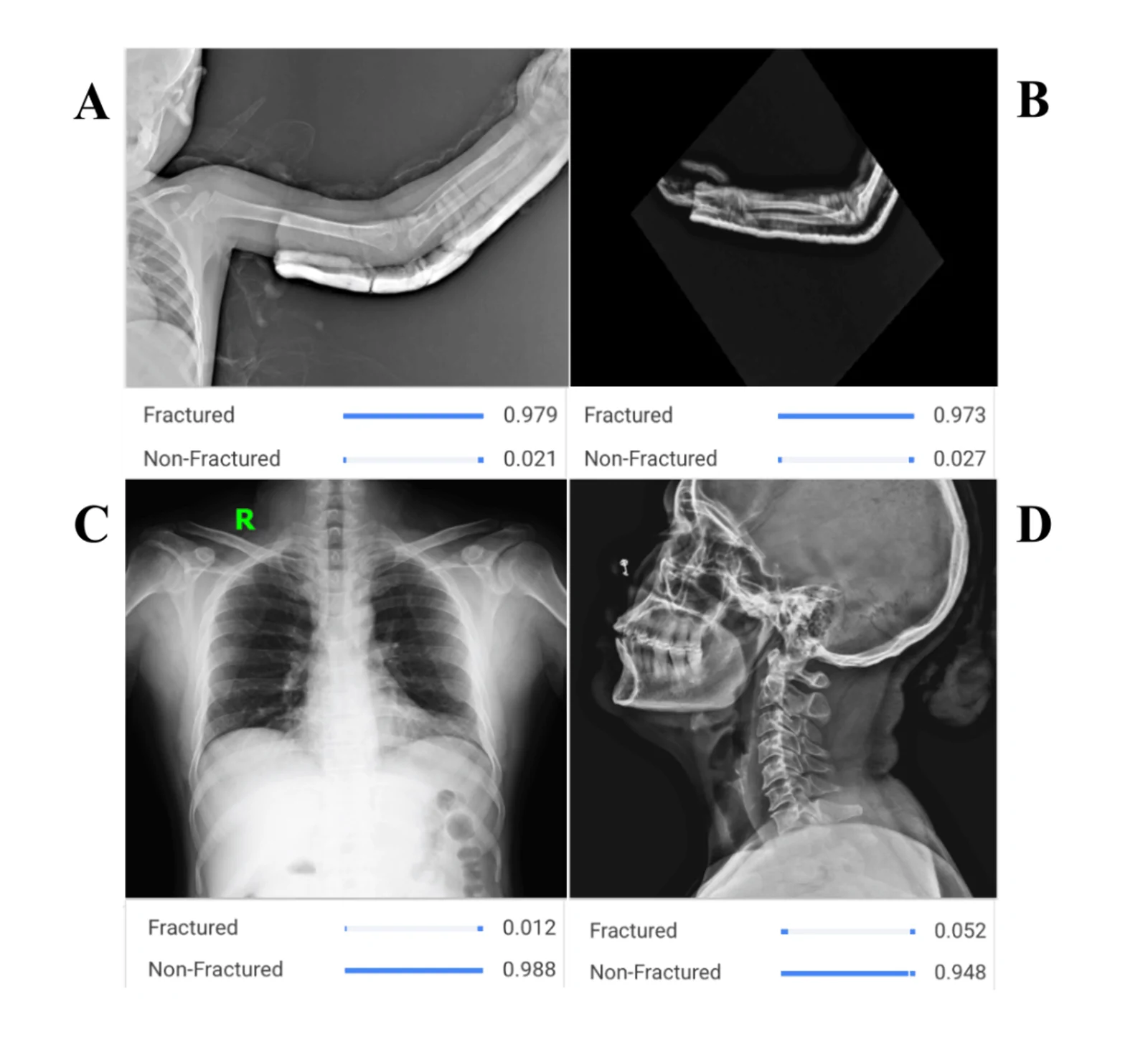
Representative Predictions Generated by the Deployed AutoML Classifier Four representative radiographs submitted to the deployed Google Cloud Vertex AI AutoML prediction endpoint are shown. Panels A and B display fractured radiographs correctly classified with fracture probabilities of 97.9% and 97.3%, respectively. Panels C and D display non-fractured radiographs correctly classified with non-fracture probabilities of 98.8% and 94.8%, respectively. These selected examples demonstrate endpoint functionality and were not treated as an independent validation set.

The trained classifier was successfully deployed to a managed online prediction endpoint within Google Cloud Vertex AI. Four representative radiographs submitted to the endpoint were classified correctly with high confidence. As shown in Figure 4, two fractured radiographs received fracture-class probabilities of 97.9% and 97.3%, whereas two non-fractured radiographs received non-fracture probabilities of 98.8% and 94.8%. These selected examples demonstrate successful online inference but do not represent an independent validation dataset or provide additional estimates of model performance.

Summary of dataset and model performance

The principal dataset characteristics, training details, and internally validated model-performance measures are summarized in Table 1.

**Table 1:** Dataset Characteristics and Internally Validated Model Performance Summary of the radiographic dataset, predefined training, validation, and test subsets, AutoML training characteristics, and internally validated classification-performance measures. Performance estimates were generated by Google Cloud Vertex AI using the platform-designated test set.

Metric	Value
Total images	420
Fractured images	130
Non-fractured images	290
Training images	336
Validation images	42
Test images	42
Evaluation type	Internal, platform-designated test set
Maximum training budget	8 node-hours
Actual training time	1 hour and 48 minutes
Confidence threshold	0.50
Overall average precision	0.987
Fracture-class average precision	0.972
Non-fracture-class average precision	0.994
Platform-reported aggregate precision	90.50%
Platform-reported aggregate recall	90.50%
Fracture sensitivity/recall	77%
Non-fracture specificity	97%
Fractures classified as non-fractured	23%
Non-fractures classified as fractured	3%

## Discussion

Principal findings

This proof-of-concept study evaluated whether a commercially available code-free automated machine-learning platform could develop and deploy a binary classifier for fractured and non-fractured bone radiographs without investigator-written code or manual neural-network design. The most clinically important finding was that the model correctly identified 77% of fractured radiographs, corresponding to approximately 23% of fractured images being misclassified as non-fractured. This limitation is more clinically informative than the overall average precision of 0.987 and underscores the need for cautious interpretation of the model’s internal test performance. The average precision value is therefore presented as a complementary threshold-independent metric rather than as the principal indicator of clinical effectiveness. However, class-specific evaluation demonstrated an important difference in performance: 97% of non-fractured radiographs were correctly classified, whereas only 77% of fractured radiographs were correctly identified. Accordingly, approximately 23% of fractured images were misclassified as non-fractured. These findings demonstrate the technical feasibility of code-free AutoML for medical-image classification while also showing that strong aggregate metrics may obscure clinically important false-negative predictions.

The trained classifier was also successfully deployed to a managed online prediction endpoint, demonstrating that a graphical AutoML workflow can extend from image import and model training to cloud-based inference. This deployment demonstrates the potential methodological utility of code-free platforms for rapid prototyping and may have future educational applications. However, educational effectiveness, usability, accessibility, learner outcomes, and educational value were not evaluated in the present study. Nevertheless, successful endpoint deployment should not be interpreted as evidence of clinical readiness because the model was evaluated using a small internal test set and was not assessed prospectively or on an independent external dataset.

Comparison with prior research

AI has demonstrated promising performance in musculoskeletal fracture detection, but prior studies have generally evaluated models using more anatomically defined datasets, larger or differently structured cohorts, and study-specific validation strategies [3-10]. In contrast, the present study used a small, heterogeneous public dataset of 420 radiographic images with limited metadata regarding anatomical site, fracture subtype, patient-level independence, and image provenance, and model performance was assessed on only 42 held-out test images. These differences substantially limit direct performance comparisons with previously published fracture-detection models and reinforce that the present findings should be interpreted primarily as a proof-of-concept evaluation of a code-free AutoML workflow rather than as evidence of competitive diagnostic performance. Lindsey et al. reported that a deep neural network improved clinician fracture-detection performance when used as a decision-support tool, while Olczak et al. demonstrated that convolutional neural networks could identify abnormalities across orthopedic trauma radiographs [5,6]. These findings support the potential role of AI as an adjunct to radiographic interpretation rather than as a replacement for clinicians.

Systematic reviews and meta-analyses have reported strong diagnostic performance for AI fracture-detection systems but have also identified important limitations, including retrospective study designs, single-source datasets, heterogeneous evaluation methods, and insufficient external validation [3,4]. These concerns are directly relevant to the present study. Although the observed average precision of 0.987 indicates strong discrimination within the study dataset, direct comparison with previously published models is limited by differences in anatomical distribution, preprocessing, dataset composition, model architecture, and validation strategy. More importantly, the reduced recall for fractured radiographs demonstrates that favorable overall performance does not necessarily translate into acceptable fracture-detection sensitivity.

Code-free AutoML and accessibility

One of the principal motivations for this study was to evaluate whether code-free AutoML could reduce the technical barriers traditionally associated with deep-learning development. Conventional machine-learning workflows may require programming proficiency, familiarity with specialized frameworks, hyperparameter optimization, access to graphics-processing hardware, and software-engineering experience [11,12]. These requirements may limit participation by clinicians, students, and biomedical researchers who possess clinical expertise but lack formal computer-science training.

Previous studies have demonstrated that automated machine-learning platforms can produce useful medical image-classification models while substantially reducing implementation complexity [13-16]. In the present study, Google Cloud Vertex AI automated image preprocessing, architecture selection, hyperparameter optimization, model training, evaluation, and deployment through a graphical interface. No custom code or investigator-directed model optimization was required. This workflow illustrates how code-free platforms may broaden participation in early-stage medical AI research.

However, accessibility does not eliminate the need for rigorous methodology. Model performance remains highly dependent on dataset quality, labeling accuracy, representative sampling, class balance, patient-level data separation, and appropriate validation. In addition, the proprietary nature of the AutoML platform limited transparency because the final neural-network architecture and several underlying training parameters were not disclosed. Code-free platforms may therefore simplify technical implementation while introducing limitations in reproducibility and interpretability.

Clinical implications

The principal implication of this study is methodological rather than clinical. The findings demonstrate that a commercially available code-free AutoML platform can be used to develop and deploy a binary radiographic image-classification model without investigator-written code or manual neural-network design. However, the present study did not evaluate clinician performance, diagnostic workflow, interpretation time, patient outcomes, or clinical decision-making. Accordingly, the results should not be interpreted as demonstrating clinical benefit.

The most clinically relevant performance finding was the fracture-class recall of 77%, indicating that approximately 23% of fractured radiographs were classified as non-fractured. A model missing approximately one in four fractured images would not be suitable for independent clinical diagnostic use. In addition, the classifier provided only a binary fractured versus non-fractured output and did not localize fractures, identify anatomical regions, characterize fracture morphology or displacement, or provide treatment recommendations. These findings therefore support technical feasibility only and not diagnostic safety, effectiveness, or readiness for clinical implementation.

Code-free AutoML may nevertheless have potential methodological utility by reducing the programming requirements associated with preliminary medical-image classification research. It may also have potential educational applications for introducing investigators or trainees to machine-learning workflows; however, usability, accessibility, educational effectiveness, learner outcomes, and educational value were not evaluated in this study. Any transition from proof-of-concept model development to clinical decision support would require substantially more rigorous validation.

Strengths

This study has several strengths. First, it demonstrates a completely code-free workflow spanning dataset import, model training, internal evaluation, deployment, and online inference. Second, the radiographic dataset is publicly accessible, allowing other investigators to examine the same source material and evaluate similar workflows. Third, class-specific performance was considered in addition to model-level summary metrics, which revealed the substantially lower recall for fractured radiographs. Fourth, the study explicitly distinguishes technical feasibility from clinical effectiveness and acknowledges important limitations related to dataset composition and validation.

The workflow itself should not, however, be considered fully reproducible in the computational sense. Google Cloud Vertex AI is a proprietary and evolving platform; the final model architecture and several internal training procedures were not disclosed, and platform behavior may change over time. Therefore, although the public dataset and general workflow can be accessed by other investigators, exact reproduction of the trained model and its performance cannot be guaranteed.

Limitations

Several substantial limitations affect interpretation of the present findings. First, model performance was evaluated using a held-out test set containing only 42 radiographic images. This is an extremely small sample from which to estimate classification performance, particularly because fractured images represent only a subset of those 42 observations. A small number of classification errors could therefore produce large changes in the reported percentages. The retained Vertex AI output provided row-normalized percentages rather than raw class-specific prediction counts, preventing reliable calculation of exact confusion-matrix counts and 95% confidence intervals. This directly limits the statistical precision, interpretability, and reproducibility of the reported performance estimates.

Second, the dataset was imbalanced, with 130 fractured images (31.0%) and 290 non-fractured images (69.0%). No investigator-directed class weighting, oversampling, undersampling, or other balancing procedure was performed. Whether the proprietary AutoML training process applied internal class weighting or sampling adjustments could not be determined from the available platform information. Because class prevalence may influence several classification metrics, the model-level results should be interpreted together with class-specific performance rather than in isolation.

Third, characterization of the underlying radiographic dataset was limited. The publicly available metadata did not provide sufficiently detailed information regarding anatomical distribution, fracture subtype, imaging projection, patient demographics, number of radiographs per patient, pathological fractures, postoperative imaging, or several other clinically relevant characteristics. Consequently, it is difficult to define precisely what spectrum of “fracture classification” was evaluated or to determine whether model performance differed by upper extremity, lower extremity, spine, or other anatomical regions. This represents a fundamental limitation because the anatomical composition of the dataset may strongly influence what features the classifier learned.

Fourth, patient-level independence could not be verified. The public documentation did not provide patient identifiers or evidence that training, validation, and test partitions were separated at the patient level. Removal of duplicate image files does not establish patient-level deduplication. If multiple radiographs from the same patient were present across different partitions, patient-specific or examination-specific characteristics could have been encountered during both training and testing, resulting in data leakage and potentially inflating the held-out test performance. This is therefore not merely a theoretical concern but a fundamental threat to the validity of the reported internal performance estimates.

Fifth, the heterogeneous nature of the dataset introduces the possibility of shortcut learning. Fractured and non-fractured images may have differed systematically in anatomical site, image positioning, cropping, borders, acquisition characteristics, or image quality. The classifier may therefore have learned dataset-specific features associated with class membership rather than fracture morphology itself. Because detailed metadata and explainability analyses were unavailable, this possibility could not be excluded.

Sixth, although the original dataset documentation states that fracture-status labels were clinically verified by a consultant surgeon, the available documentation does not describe the reference-standard process in sufficient detail to determine whether independent radiologist review, multiple-reader consensus, or formal orthopedic adjudication was performed. The investigators did not independently relabel or adjudicate the images. Uncertainty regarding the reference standard therefore remains a potential source of label error.

Seventh, the study relied on a Kaggle derivative of the original Mendeley dataset. Although the derivative was selected because it provided a machine-learning-ready image collection, image-for-image equivalence with the original source and the precise origin of the training, validation, and test partition could not be independently verified. This limits complete dataset provenance and reproducibility.

Eighth, no independent external validation was performed. Performance obtained from a small held-out subset of the same underlying public dataset cannot establish generalizability to radiographs from different institutions, imaging systems, acquisition protocols, patient populations, or clinical environments [19,20]. The four endpoint examples were included only to demonstrate inference functionality and should not be interpreted as additional validation evidence.

Finally, the model performed only binary image-level classification. It did not localize fractures, distinguish fracture morphology or anatomical region, evaluate multiple simultaneous abnormalities, assess calibration or subgroup fairness, or compare performance with radiologists, orthopedic surgeons, emergency physicians, or custom-developed deep-learning models. Clinical workflow effects, interpretation time, prospective diagnostic performance, and patient outcomes were also outside the scope of this proof-of-concept study.

Future directions

Future investigations should address these limitations using substantially larger, independently curated, multicenter radiographic datasets with confirmed patient-level separation between training, validation, and test cohorts. Detailed metadata should include anatomical region, fracture type, patient demographics, imaging projection, acquisition equipment, institutional source, and relevant clinical characteristics. Dataset construction should also explicitly evaluate duplicate patients and potential sources of shortcut learning.

Raw prediction outputs should be retained to permit calculation of exact confusion-matrix counts, sensitivity, specificity, PPV, NPV, accuracy, AUROC, precision-recall metrics, calibration measures, and 95% confidence intervals. Approaches to class imbalance, including weighting or sampling methods, should be prospectively documented.

Independent external validation is essential before conclusions regarding generalizability can be made. Future studies could additionally evaluate fracture localization, object detection, segmentation, multiclass anatomical classification, explainability techniques, and prospective clinician-AI collaboration. Although these analyses were not feasible in the present proof-of-concept study because of the limitations of the available public dataset and retained platform outputs, they represent necessary steps for determining whether code-free AutoML approaches can progress beyond technical feasibility.

The findings support the technical feasibility of using code-free AutoML to develop and deploy a binary radiographic image-classification workflow without custom programming. However, the 77% fracture-class recall, extremely small 42-image held-out test set, and absence of external validation substantially limit conclusions regarding diagnostic performance or broader utility. The model correctly identified only 77% of fractured radiographs, and interpretation of all performance estimates is constrained by the extremely small 42-image test set, class imbalance, incomplete anatomical and patient-level metadata, uncertain patient-level separation, potential shortcut learning, limited raw performance data, and absence of external validation. The findings therefore support the technical feasibility and potential methodological utility of code-free AutoML for preliminary medical-imaging research but do not establish clinical effectiveness or educational effectiveness. Rigorous patient-level and external validation would be required before clinical implementation could be considered.

## Conclusions

This proof-of-concept study demonstrates that a commercially available code-free AutoML platform can be used to develop and deploy a binary classifier for fractured and non-fractured bone radiographs without custom programming or manual neural-network design. The model achieved strong internal discrimination, with an overall average precision of 0.987; however, fracture-class recall was 77%, indicating that a clinically important proportion of fractures remained undetected. The small internal test set, class imbalance, limited clinical metadata, uncertain patient-level independence, and absence of external validation substantially limit generalizability. Therefore, the present findings support the use of code-free AutoML for rapid prototyping, education, and early-stage medical imaging research, but they do not establish clinical readiness. Larger, multicenter studies with confirmed patient-level separation, detailed metadata, and independent external validation are required before this approach can be considered for clinical implementation.
